# Transmission of Methicillin-Resistant Staphylococcus aureus to Human Volunteers Visiting a Swine Farm

**DOI:** 10.1128/AEM.01489-17

**Published:** 2017-11-16

**Authors:** Øystein Angen, Louise Feld, Jesper Larsen, Klaus Rostgaard, Robert Skov, Anne Mette Madsen, Anders Rhod Larsen

**Affiliations:** a Department of Bacteria, Parasites and Fungi, Statens Serum Institut, Copenhagen, Denmark; b National Research Centre for the Working Environment, Copenhagen, Denmark; c Department of Epidemiology Research, Statens Serum Institut, Copenhagen, Denmark; University of Helsinki

**Keywords:** MRSA, human volunteers, swine, transmission

## Abstract

Transmission of methicillin-resistant Staphylococcus aureus (MRSA) from animals to humans is of great concern due to the implications for human health and the health care system. The objective was to investigate the frequency and duration of MRSA carriage in human volunteers after a short-term exposure in a swine farm. The experimental study included 34 human volunteers staying 1 h in a MRSA-positive swine farm in four trials. In two of the trials, the influence of farm work involving pig contact was studied using a crossover design. The quantities of MRSA in nasal swabs, throat swabs, and air samples were measured at different time points and analyzed in relation to relevant covariates. This investigation showed that, overall, 94% of the volunteers acquired MRSA during the farm visit. Two hours after the volunteers left the stable, the nasal MRSA count had declined to unquantifiable levels in 95% of the samples. After 48 h, 94% of the volunteers were MRSA-negative. Nasal MRSA carriage was positively correlated to personal exposure to airborne MRSA and farm work involving pig contact and negatively correlated to smoking. No association was observed between MRSA carriage and face touching behavior, nasal methicillin-susceptible Staphylococcus aureus (MSSA) carriage, age, or gender. The increase in human MRSA carriage among the volunteers with pig contact seems to be dependent on the increased concentration of airborne MRSA of the surrounding air and not directly on physical contact with pigs. MRSA was not detected in any of the throat samples.

**IMPORTANCE** The experimental approach made it possible to elucidate the contributions of airborne MRSA levels and farm work to nasal MRSA carriage in a swine farm. Short-term exposure to airborne MRSA poses a substantial risk for farm visitors to become nasal carriers, but the carriage is typically cleared within hours to a few days. The risk for short-term visitors to cause secondary transmissions of MRSA is most likely negligible due to the observed decline to unquantifiable levels in 95% of the nasal samples after only 2 h. The MRSA load in the nose was highly correlated to the amount of MRSA in the air and interventions to reduce the level of airborne MRSA or the use of face masks might consequently reduce nasal contamination.

## INTRODUCTION

Methicillin-resistant Staphylococcus aureus (MRSA) was described for the first time in 1961 and was for several decades almost exclusively found in humans. In 2005, a new variant of MRSA belonging to clonal complex 398 (CC398) was first described in pigs and pig farmers in France and the Netherlands (1, 2). MRSA CC398 was later disseminated in pig production worldwide but is also found in other livestock animals, such as poultry and veal calves (3, 4), which has led to the designation livestock-associated MRSA (LA-MRSA). LA-MRSA CC398 is characterized by being mostly negative for the human-associated immune evasive gene cluster (containing *sea*, *scn*, *sak*, and *chp*) and Panton-Valentine leukocidin (PVL), which in contrast are found in the human CC398 MRSA lineage (5).

Transmission of LA-MRSA from animals to humans has been of great concern in some European countries, especially those with low MRSA incidence and large pig productions (e.g., Denmark), due to negative implications for human health and the health care system. Several studies have reported an increased risk for being colonized or infected with LA-MRSA among persons working in the livestock industry (6–8), but infection rates of LA-MRSA are also increasing among the general public (9, 10).

There has been a steep increase in LA-MRSA cases in Denmark since 2004, primarily in persons with swine farm contact. Since 2012, patients with regular contact with livestock have been tested for MRSA carriage at hospital admission and isolated until negative MRSA results were confirmed. However, 33% of LA-MRSA infections in Denmark are not associated with livestock contact (9). In order to diminish spread to the general public, attempts to restrict LA-MRSA to stables have been made, e.g., by improved hygiene routines for farm workers. Furthermore, there is an ongoing discussion among professionals and in the public regarding the risk of carriage in relation to short-term visits, e.g., those by school classes, veterinarians, and workmen.

The transmission routes between pigs and humans in swine farms have not yet been fully elucidated, but transmission is likely to be associated with both the within-herd MRSA prevalence (11) and the intensity of animal contact (3, 12). Hands are generally suspected to be the main vector for transmitting S. aureus from surfaces to the nose (13). However, the presence of LA-MRSA at high levels in air samples from pig farms (14–16) indicates that aerosols and contaminated dust particles may also be important in the transmission to workers and visitors (17, 18). The relative importance of physical contact and airborne transmission in human carriage of LA-MRSA is not clear and further investigations are needed to guide rational interventions to protect farm workers and visitors from becoming contaminated and reduce the risk for subsequent transmission and infection. It is of special interest to establish if certain work-related procedures carry an increased risk of contamination, as well as to establish the relationship between the LA-MRSA level in air and the degree of human MRSA carriage.

To answer these questions, we conducted a study where MRSA-negative human volunteers visited an LA-MRSA-positive swine farm. The primary objectives were to investigate the frequency and duration of MRSA carriage after short-term exposure to MRSA in a swine farm and to determine the impacts of work-related activity and associated risk factors.

## RESULTS

### Summary statistics for human volunteers.

In total, 34 volunteers were enrolled in the study. The age distribution was as follows: 20 to 29 years (*n* = 29), 30 to 39 years (*n* = 1), 40 to 49 years (*n* = 2), and ≥50 years (*n* = 2). In trials 1 to 3, 24 volunteers participated, whereas 22 participated in trial 4. The data set therefore contained 94 observations in total. Twenty-two volunteers participated in both trials 1 and 2, 12 volunteers participated in all 4 trials, 10 volunteers participated 3 times, four volunteers participated twice, and eight volunteers participated in one of the trials. Seven volunteers (22%) were male and seven volunteers (22%) were smokers (6 females and 1 male).

### MRSA carriage in human volunteers.

**FIG 1:**
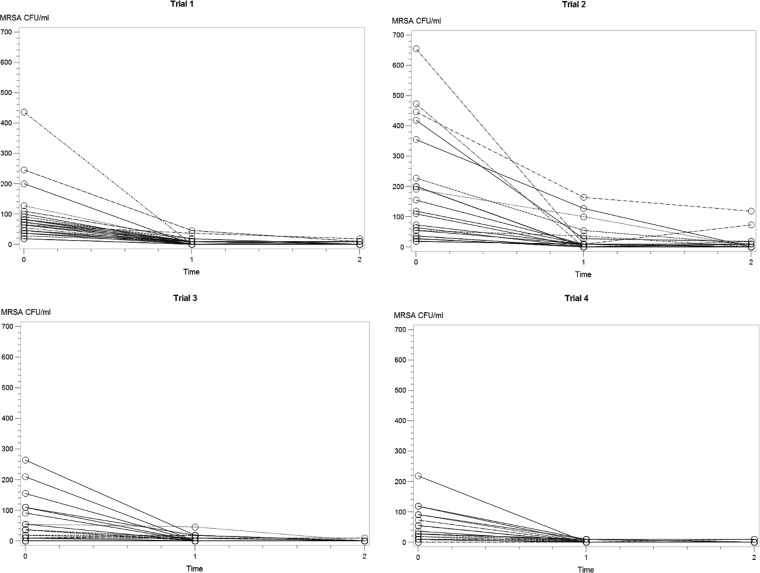
Nasal MRSA count (CFU/ml swab fluid) of human volunteers 0, 1, and 2 h after leaving the stable, shown for the four trials performed.

All samples taken before entering the swine farm were MRSA-negative. In total, 88/94 (94%) of the volunteers became positive after the 1-h stay in the stable. During the visits with high MRSA load in the air (trials 1 and 2), the nasal samples of all 48 volunteers were MRSA-positive when leaving the stable (time [T] = 0). In trials 3 and 4, where all volunteers were passive observers and where the load of MRSA in the air was relatively low, 6/46 (13%) of the volunteers were MRSA-negative when leaving the stable. All strains were confirmed as LA-MRSA CC398 with *spa* type t011. In all trials, a sharp decline in MRSA count was observed during the first 2 h after leaving the stable (Fig. 1; see also Table S1 in the supplemental material), and after 2 h the amount of MRSA was unquantifiable in 95% of the samples (growth of ≤1 MRSA colony by direct plating or only positive after enrichment). The number and percentage of MRSA-positive volunteers after different time points are shown in Table 1 (see additional details in Table S1). After 48 h, 94% of the nasal swabs were MRSA-negative; in trials with high versus low MRSA load in the air, the corresponding values were 90% and 98%, respectively. Only one volunteer was MRSA-positive after 7 days, but tested negative on day 14. All participants in trials 1 and 2 were still MRSA-negative after 3 weeks when enrolling to the next trial. MRSA was not detected in any of the throat samples at any time. Summary statistics on class variables in relation to MRSA counts from the volunteers at different time points can be found in Table S2 in the supplemental material.

**TABLE 1:** MRSA-positive^*a*^ volunteers after farm visit

Trial no.^*b*^	No. of volunteers	No. (%) of MRSA-positive volunteers according to time after leaving stable					
		0 h	1 h	2 h	1 day	2 days	7 days
1	24	24 (100)	13 (54)	5 (21)	1 (4)	1 (4)	0
2	24	24 (100)	18 (75)	12 (50)	4 (17)	4 (17)	1 (4)
3	24	19 (79)	11 (46)	3 (13)	2 (8)	1 (4)	0
4	22	21 (95)	8 (36)	2 (9)	3 (14)	0 (0)	0
All trials	94	88 (94)	50 (53)	22 (23)	10 (11)	6 (6)	1 (1)

a After enrichment culture.

b Trials 1 and 2, high MRSA exposure in the stable; trials 3 and 4, low MRSA exposure in the stable.

### Methicillin-susceptible Staphylococcus aureus carriage.

Thirteen of the volunteers (41%) had a persistent nasal methicillin-susceptible Staphylococcus aureus (MSSA) population and were accordingly recorded as MSSA carriers. Nineteen of the volunteers (59%) were persistent MSSA throat carriers. Seven volunteers (21%) were MSSA carriers in both the nose and throat and identical *spa* types were found in both locations. Seven volunteers (21%) were MSSA-negative in both the nose and throat in all trials.

### Exposure to MRSA in air.

**FIG 2:**
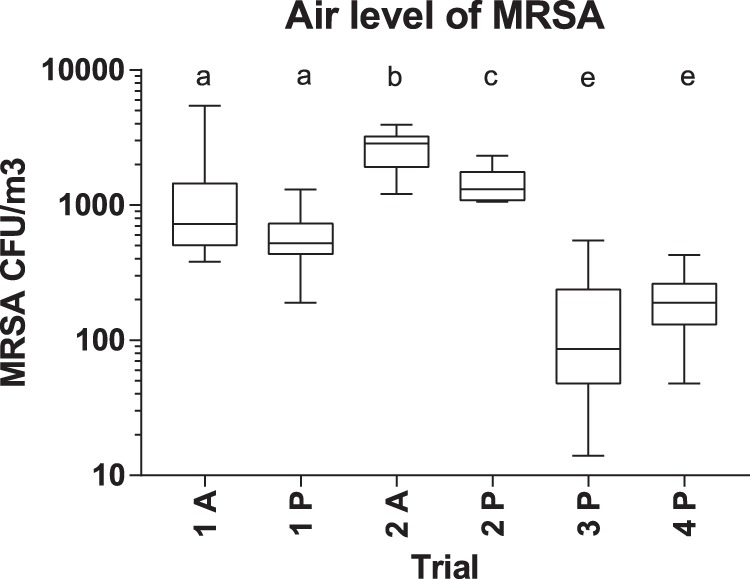
Air level of MRSA (CFU/m^3^) in the four trials. A, active group with pig contact; P, passive group. Box-and-whisker plot indicates median, interquartile ranges, and maximum and minimum values. Different letters above the graph indicate significant differences between groups (two-sided *t* test, *P* < 0.05).

Personal exposure to airborne MRSA varied between 24 and 5,452 CFU MRSA/m^3^ (geometric mean [GM] = 384; standard deviation [SD] = 1,033). The exposure level was significantly higher in trials 1 and 2 (GM = 1,133; SD = 1,116) than in trials 3 and 4 (GM = 124; SD = 131) which was as expected according to the initial investigations in the herd. There was a significant difference in exposure levels between trials 1 and 2, but not between trials 3 and 4. Being in the active group also had an effect on the personal exposure levels of the volunteers in trials 1 and 2, with the highest exposure in the groups taking pig samples. However, the difference was only statistically significant in trial 2 (Fig. 2). The exposure levels on the different days of the trials are shown in Fig. S1 in the supplemental material. The volunteers in the active group had the highest MRSA carriage levels at all time points after the farm visit (see Fig. S2 and S3 in the supplemental material).

**FIG 3:**
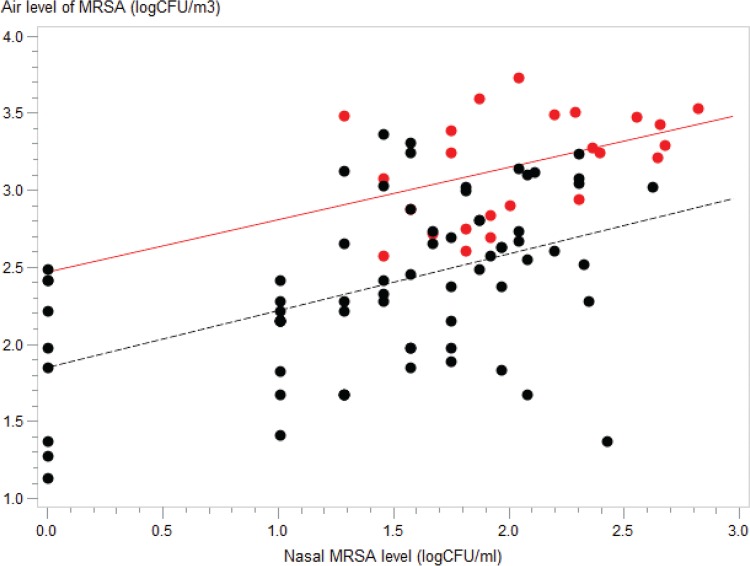
Correlation between nasal MRSA level immediately after leaving the stable and MRSA level in the air. Data points corresponding to active volunteers with pig contact in trials 1 and 2 are marked in red. Black points correspond to passive volunteers in all four trials. Regression lines are indicated for the active group (y = 2.46 + 0.34*x*, coefficient of determination [*R*^2^] = 0.18) and passive group (y = 1.85 + 0.37*x*, *R*^2^ = 0.22). The regression line for all data points combined (not shown) corresponded to y = 1.78 + 0.5*x* (*R*^2^ = 0.33).

There was a correlation between the nasal MRSA level immediately after leaving the stable and personal exposure to airborne MRSA (Fig. 3; data points corresponding to volunteers in the active group are marked in red). The correlation between the nasal MRSA level and personal exposure to airborne MRSA had almost disappeared by 1 and 2 h after leaving the stable (see Fig. S2).

The six volunteers who tested MRSA-negative immediately after leaving the stable in trials 3 and 4 had all been exposed to air levels in the range of 24 to 310 CFU MRSA/m^3^ (GM = 66; SD = 131). On the other hand, five volunteers that had been exposed to similar concentrations were still positive after 24 h; one of these was also MRSA-positive after 48 h.

### Multivariable analysis.

The effects of pig contact and face touching on nasal MRSA carriage were estimated using data from trials 1 and 2 (see model A, Table 2). Among the volunteers with pig contact, the number of face touches in trials 1 and 2 was on average 5.6 per hour (range 1 to 32; SD = 6.2). The multivariable analysis showed that nasal MRSA carriage level was dependent on (i) the level of exposure to airborne MRSA and (ii) whether the person was in the active group during the visit. According to the model, a doubling of the airborne MRSA level corresponded to a 73% increase in the nasal MRSA carriage level among the volunteers without pig contact and to a 168% increase in MRSA carriage among volunteers in the active group. Nonsignificant factors in this analysis were age, gender, smoking habit, the number of face touches, and nasal MSSA carriage. The random between-person variation in carriage level (highest versus lowest of two levels) had a median ratio of 1.67.

**TABLE 2:** Determinants of MRSA carriage level in nasal swabs according to multivariable analysis

Model^*a*^	Variable	Relative carriage level (CI)^*b*^
A	Exposure level of airborne MRSA^*c*^	1.73 (1.70–1.77)
	Farm work (active)^*d*^	1.55 (1.59–1.60)
	Farm work (passive)^*e*^	1
B	Exposure level of airborne MRSA^*c*^	1.42 (1.39–1.45)
	Smoker	0.44 (0.21–0.92)
	Nonsmoker^*e*^	1

a Model A, trials 1 and 2; model B, trials 1 to 4, excluding volunteers with pig contact.

b The confidence interval (CI) for each value is shown in parentheses.

c Exposure was measured in log_2_-transformed CFU/m^3^.

d Farm work with pig contact.

e Reference.

The factors influencing nasal MRSA carriage of the volunteers were subsequently analyzed, including only passive observers from all four trials. This analysis showed that nasal MRSA carriage level was (i) positively correlated to personal exposure to airborne MRSA, whereas it was (ii) negatively correlated to smoking (see model B, Table 2). According to this model, a doubling of the airborne MRSA level corresponded to a 42% increase in nasal MRSA carriage. Smoking reduced nasal MRSA carriage by 56% compared to nonsmokers. Nonsignificant factors in this analysis were age, gender, and MSSA carriage. The random between-person and between-trial variations in carriage level had median ratios of 1.76 and 1.10, respectively.

## DISCUSSION

### Acquisition and loss of MRSA.

This study showed that the main determinants for nasal MRSA carriage among the volunteers were the personal exposure level to airborne MRSA, performing work with pig contact, the time passed since leaving the stable, and smoking habit. These factors have all previously been reported to be related to MRSA carriage. Several studies found MRSA in aerosols from swine farms (14–16) and one study showed that an increased level of MRSA in the air is correlated to increased MRSA carriage in humans (17).

The present study included a detailed investigation of nasal MRSA carriage in the hours following a farm visit. In total, 94% of the volunteers were MRSA-positive when leaving the stable, but the amount of bacteria was reduced to unquantifiable levels in 95% of the samples when measured 2 h after leaving the stable. Thus, due to the short duration of the observed MRSA carriage, it is more correct to regard this as a transient contamination. Nevertheless, 12% were still MRSA-positive after 24 h and 6% were MRSA-positive after 48 h. This is higher than what was reported in a Dutch study where only 6% were positive after 24 h (10). However, in that study only 44% of the participants were MRSA-positive when leaving the stable and a lower level of MRSA exposure may therefore explain the difference. Another study that included 30 veterinary students reported that 22% were MRSA-positive after visits to MRSA-positive swine farms and that all were MRSA-negative 24 h later (19). The MRSA air levels were not measured in these two studies, so a direct comparison to our results is difficult.

### Transmission dynamics.

Several investigations have previously tried to elucidate the transmission dynamics by using indirect measures for physical contact, e.g., working time and work location (12, 17), but it has been difficult to produce conclusive evidence on the different transmission routes by observational studies alone. In the present study, farm work (the active group) was defined as 1 h of active sampling of nasal and skin swabs from swine. The volunteers worked in groups of three, alternating regularly between the tasks of catching and constraining pigs, taking samples, and supportive functions, thereby ensuring a homogenous exposure within the group. The volunteers in the active group had a significantly increased airborne exposure and nasal carriage level of MRSA compared to the passive volunteers. Direct physical transfer between the hands and the face could not explain the higher nasal carriage level in the active group.

These findings suggest that the direct contact with pigs is not the determining factor by itself and that the increase in human MRSA contamination is mainly a function of the increased concentration of airborne MRSA in the surrounding air. This is supported by the multivariable analyses, where a doubling of the exposure to airborne MRSA corresponded to a higher increase in the nasal MRSA contamination level among the volunteers in the active group (186%) than in the passive group (73% in trials 1 and 2 [model A] and 42% in trials 1 to 4 [model B], respectively). An increased respiratory rate can be expected among the volunteers in the active group, which might explain the higher nasal deposition of MRSA compared to the passive group. An increased nasal MRSA deposition can therefore be expected also in connection with other forms of farm work generating dust or increased physical activity.

### Covariates.

Smoking was associated with a lower level of MRSA contamination among the volunteers. A reduced S. aureus carriage level associated with smoking has also been reported by others (3, 20).

MRSA contamination was not associated with age, gender, or MSSA carriage in our study. The limited age range among the volunteers and the fact that mainly female subjects participated make it difficult to draw conclusions from this study regarding the significance of these parameters.

In the present study, we divided the volunteers into persistent MSSA carriers and intermittent/noncarriers according to recommendations by van Belkum et al. (21); MSSA status was not found to be associated with MRSA carriage. Earlier investigations have indicated that carriage of MSSA might have a protective effect against colonization of MRSA (22). On the other hand, there are reports indicating that nasal carriage of S. aureus predisposes subjects to rather than protects against staphylococcal acquisition in the nose (23).

### MRSA contamination in the nose but not in the throat.

The anterior nasal cavity is the main colonizing site for S. aureus in humans (18, 24). A persistent MSSA population was found in the nose and the throat of 41% and 59% of the volunteers, respectively. In the present study, MRSA was found as a transient contaminant of the nasal cavity, whereas the throat swabs collected during the trials were all MRSA-negative. This might indicate that colonization of the throat does not take place directly from inhaled air. In accordance with the present study, other investigations have shown that simultaneous carriers have identical S. aureus genotypes in their nose and throat, indicating that the throat is colonized following establishment in the posterior part of the nasal cavity (25).

### Quantification of airborne MRSA.

The air level of MRSA measured by personal Gesamtstaubprobenahme (GSP) samplers showed a significant correlation between the MRSA levels in the nose and air (Fig. 3). There were, however, many deviating observations, which indicate that individual factors have great importance when determining the carriage level of each individual. This random between-person variation represents a challenge in regard to determining a colonizing dose for MRSA. Only six volunteers were MRSA-negative when leaving the stable (Table 1). All volunteers exposed to air levels above 310 CFU/m^3^ were MRSA-positive when leaving the stable. However, other volunteers exposed to MRSA levels in the range of 24 to 310 CFU/m^3^ were MRSA-positive for up to 2 days after leaving the stable. It was therefore not possible to determine a general and specific colonizing dose for MRSA.

### Sampling limitations.

Repeated sampling of the nasal cavity might influence the MRSA count by physically removing parts of the bacterial population, thus confounding an accurate assessment of bacterial loss. Other factors affecting the amount of nasal mucus, e.g., nasal secretion and sneezing induced by the farm visit, can also be expected to have impact on repeated nasal sampling. Only the anterior part of the nasal cavity was sampled in this study. Nasal lavage could have assessed a larger part of the MRSA population present in the nasal cavity but would probably have had an even higher confounding effect upon repeated sampling.

GSP samplers are commonly used for dust sampling and have also been used for MRSA sampling (12). However, loss of viability of vegetative cells may occur, presumably due to desiccation stress during sampling (26), leading to an underestimation of the number of airborne bacteria.

### Study limitations.

The strength of this study is the experimental approach whereby it was possible to analyze the relative importance of work, direct contact with pigs, and air transmission for human MRSA contamination after a short-term visit to a swine farm. The study included data from a single swine farm and a single LA-MRSA strain and was based on MRSA-negative, healthy volunteers, primarily between 20 and 30 years old. In spite of these limitations, the conclusions can probably be extrapolated to several other groups, e.g., craftsmen, veterinarians, school classes, and other short-term visitors. However, the results may not be directly applicable to farm workers spending longer durations in swine farms, as longer colonization times have been observed in this group (27).

### Public health significance.

This study shows that short-term visitors to MRSA-positive swine farms will experience transient contamination of the nasal cavity. The risk for short-term visitors to cause secondary transmissions of MRSA is most likely negligible, due to the observed decline to unquantifiable levels in 95% of the nasal samples after only 2 h.

The MRSA load in the nose was highly correlated to the amount of MRSA in the air and interventions to reduce the level of airborne MRSA or the use of face masks might consequently reduce the nasal contamination.

### Conclusions.

This study has shown that the nasal MRSA contamination level is positively correlated to the air level of MRSA and to farm work, and negatively correlated to the time passed since leaving the farm and smoking habit. No association was observed between MRSA contamination and face touching behavior, nasal MSSA carriage, age, or gender. An increased level of airborne MRSA had a higher impact on nasal MRSA contamination among volunteers performing farm work than on passive volunteers. This probably reflects the higher respiratory activity of active volunteers. MRSA contamination declined quickly after the farm visit and after 48 h MRSA could only be detected in 6% of the volunteers.

## MATERIALS AND METHODS

### Recruitment of volunteers.

Human volunteers were recruited through advertisements at the University of Copenhagen. Most of the volunteers were students within the fields of veterinary science and animal husbandry. In addition, a few staff members from the Statens Serum Institut participated. All volunteers participated in an information meeting, received written project information, and signed a declaration of informed consent. The participants filled in a questionnaire and were tested for MRSA carriage in the nose and throat. Subjects were eligible for participation if they (i) were healthy individuals above 18 years of age, (ii) tested negative for MRSA in the nose and throat, (iii) did not have professional exposure to swine, (iv) did not work in health care facilities, (v) did not have allergies to dust, (vi) had not used antibiotics during the last 3 months, and (vii) did not have skin diseases or wounds. The study was performed in accordance with the principles of the Declaration of Helsinki and was approved by the National Committee on Health Research Ethics (Protocol H-15013814).

### Study design.

The study was conducted on an LA-MRSA-positive swine farm. We used the weaner section for our study, as initial investigations showed a low MRSA load the first week after weaning and a high MRSA load approximately 4 weeks after weaning, making it feasible to conduct experiments with different MRSA loads. The farm was visited in 2016 on two consecutive days in each of the weeks 14, 17, 21, and 39, here referred to as trials 1 to 4. In trials 1 and 2, we visited stables with pigs approximately 4 weeks after weaning (high MRSA exposure), whereas in trials 3 and 4, we visited stables with pigs 1 to 2 weeks after weaning (low MRSA exposure).

Twelve and 11 volunteers visited the stable on each day in trials 1 to 3 and in trial 4, respectively. The influence of farm work with physical contact with pigs during the farm visit on subsequent MRSA carriage was studied in trials 1 and 2 using a crossover design. Six volunteers worked in groups of three persons inside the pens by taking nose and skin swabs from the pigs for 60 min, here referred to as the active group. Meanwhile, six volunteers in the passive group stayed in the corridor separating the pens and were instructed not to touch anything in the room. During the 60 min of the study, each member of the passive group registered the number of hand-to-face skin touches for one of the active volunteers. The volunteers allocated to the active group in trial 1 constituted the passive group in trial 2 and vice versa. In trials 3 and 4, all volunteers were passive observers and stayed in the corridor between the pens for 60 min without touching anything in the room.

### Procedures at the farm.

Before entering the farm, all volunteers washed their hands and changed clothes. The volunteers were dressed in a clean pair of boots and a disposable suit (Tyvek Classic Xpert, DuPont) covering the whole body, including the hair and leaving only the hands and the frontal part of the face exposed. When leaving the farm, the volunteers changed clothes and washed their hands. They were not allowed to smoke or wash their face within the first 2 h after leaving the stable.

### Human sampling.

Swab samples were taken from the nose and throat of the volunteers using eSwab (Copan). Nasal samples were taken from the anterior part of the nose by rotating the same swab 5 times in each nostril. Throat samples were obtained by swabbing the palatopharyngeal arch and tonsils on both sides. Nasal and throat samples were taken 2 h before entering the stables and immediately after leaving the stable (T = 0). In addition, nasal samples were taken 1 and 2 h after leaving the stable in all trials, whereas corresponding throat samples were only collected in trial 1, as all throat samples turned out to be MRSA-negative. All samples were kept in a cooling box and cultivation was initiated approximately 3 h after leaving the stable. Additional nasal samples were taken the day after the visit (day 1), nasal and throat samples were obtained on day 2, and a final nasal swab was taken on day 7. If MRSA was detected in the final sample, an additional nasal sample was obtained 14 days after the farm visit. All samples were taken by the principal investigator except the nasal samples on day 1 and 7, which were taken by the volunteers themselves. The volunteers kept these samples at 4°C for up to 24 h before cultivation was initiated.

### Microbiological and molecular analyses of human samples.

From each sample, MRSA was quantified by making serial dilutions of the swab fluid (1 ml) with 0.9% NaCl added to 0.1% Triton X-100 (Sigma-Aldrich), followed by spread of 100 μl on one Brilliance MRSA 2 agar plate (Oxoid) and incubation at 35°C for 22 to 24 h. Furthermore, all samples were investigated for MRSA by enrichment in tryptic soy broth (Sigma-Aldrich) supplemented with 6.5% NaCl at 35°C for 16 to 24 h, followed by spread of 10 μl on Brilliance MRSA 2 agar plates and incubation at 35°C for 22 to 24 h. MRSA was identified as denim blue colonies. One colony from each volunteer at T = 0 was selected for molecular verification. In addition, one colony from each person showing growth of presumptive MRSA-colonies on day 1 or later, as well as all colonies showing an atypical phenotype, was verified by PCR.

Methicillin-sensitive S. aureus (MSSA) was detected on SaSelect agar plates (Bio-Rad) as pink colonies after cultivation at 35°C for 22 to 24 h. Four presumptive S. aureus colonies from each volunteer were subcultivated on 5% blood agar plates for subsequent molecular analysis. The presence of MSSA was investigated from nasal and throat samples taken before and 2 days after the farm visit as well as from throat samples collected at T = 0, as initial investigations had shown that these contained predominantly MSSA.

All MRSA and MSSA subcultures were verified by a PCR assay detecting *mecA*, *lukF-PV*, *scn*, and *spa*, followed by *spa* typing (28). MRSA was identified by the presence of *mecA* and *spa* amplicons. The presence of only a *spa* amplicon was indicative for MSSA.

Based on *spa* typing of the four MSSA isolates from each sample, the persistence of MSSA carriage of the nose or the throat was assessed. A carrier was defined as a person having MSSA with identical *spa* types in the majority (>80%) of the sampling events; the other participants were recorded as noncarriers (including transient carriers) following the recommendations by van Belkum et al. (21).

### Personal exposure sampling of airborne particles.

Airborne MRSA was sampled using GSP (Gesamtstaubprobenahme) samplers (CIS by BGI, Inc., Waltham, MA, USA). The GSP samplers with a Teflon filter (pore size 1 μm; Millipore, Bedford, MA, USA) were mounted in the inhalation zone on the chest and were connected by flexible tubes to a pump adjusted to an airflow of 3.5 liters/min. In each trial, sampling was performed for approximately 60 min during the stay in the stable. The exact sampling time for each person was noted and used for calculation of exposure (CFU/m^3^ air). The GSP samplers were unmounted outside the stable before leaving the farm, and all samplers were transported in closed boxes to the laboratory within 2 h after sampling for quantification of MRSA.

### Microbiological and molecular analyses of air samples.

Extraction of dust particles was performed immediately after arrival at the laboratory as previously described (29). Briefly, filters were extracted in 5 ml liquid (0.85% NaCl added to 0.05% Tween 80) by orbital shaking (500 rpm) for 15 min at room temperature. MRSA was quantified by spreading 500 μl of the dust suspension in duplicate on Brilliance MRSA 2 agar plates. The agar plates were incubated at 37°C for 22 to 24 h and MRSA colonies were identified by denim color and counted. The only two negative air samples (both from trial 3) were reexamined and MRSA was detected after recultivation from frozen samples. The MRSA content of these two samples was set to the detection limit of the test (24 CFU/m^3^).

For verification of MRSA, five randomly selected isolates from each sampling day were subjected to PCR analysis for *mecA*, *lukF-PV*, *scn*, and *spa*, followed by *spa* typing (28).

### Data collection.

The following data regarding the volunteers were extracted from the questionnaire: age, gender, and regular smoking habit. During the farm visits in trial 1 and 2, allocation to group (active or passive role) and the number of face touches for the active group were recorded. The quantity of MRSA per ml of swab fluid was recorded at T = 0 as well as at 1 and 2 h after leaving the farm. In addition, the detection of MRSA after enrichment was recorded for all samples. The amount of MRSA (CFU/m^3^) in the air was obtained from the personal GSP samplers.

### Statistical analysis.

All statistical analyses were performed with SAS Enterprise 6.1 (SAS Institute, Inc.) and GraphPad Prism 7 (GraphPad Software, Inc.). A multivariable analysis was performed using the MRSA counts per ml swab fluid when leaving the stable as the dependent variable. A multivariable model was evaluated using the GLIMMIX procedure (based on the Poisson distribution) and using person identifier (ID) as random effect. The estimated size of the random effect was presented using the median ratio parameter according to Larsen et al. (30). The airborne MRSA level was log_2_ transformed in order to facilitate a direct interpretation relative to the nasal MRSA carriage. Models with and without interaction terms were evaluated. When estimating the effect of pig contact and face touching on MRSA carriage, only data from trials 1 and 2 were used (model A). In addition, a model was evaluated using data from all 4 weeks, excluding the volunteers that had pig contact in trials 1 and 2 (model B). In this model, both trial number and person ID were included as random effects. The best model fit and hence the models to be analyzed and presented were found by a combined forward and backward selection process by selecting significant variables (*P* < 0.05). For model A, an interaction term could not be fitted due to quasiseparation of data. A scatter plot was made of the log-transformed values of airborne MRSA and MRSA carriage of the volunteers (MRSA values were added to 1 before transformation).

## Supplementary Material

Supplemental material

## References

[1] Armand-Lefevre L, Ruimy R, Andremont A 2005 Clonal comparison of *Staphylococcus aureus* isolates from healthy pig farmers, human controls, and pigs. Emerg Infect Dis 11:711–714. doi:10.3201/eid1105.040866. 15890125 PMC3320358

[2] Voss A, Loeffen F, Bakker J 2005 Methicillin-resistant *Staphylococcus aureus* in pig farming. Emerg Infect Dis 11:1965–1966. doi:10.3201/eid1112.050428. 16485492 PMC3367632

[3] Graveland H, Wagenaar JA, Heesterbeek J, Mevius D, Van Duijkeren E, Heederik DJJ 2010 Methicillin resistant *Staphylococcus aureus* ST398 in veal calf farming: human MRSA carriage related with animal antimicrobial usage and farm hygiene. PLoS One 5:e10990. doi:10.1371/journal.pone.0010990. 20544020 PMC2882326

[4] van Duijkeren E, Hengeveld P, Zomer TP, Landman F, Bosch T, Haenen A, van de Giessen A 2016 Transmission of MRSA between humans and animals on duck and turkey farms. J Antimicrob Chemother 71:58–62. doi:10.1093/jac/dkv313. 26490016

[5] Price LB, Stegger M, Hasman H, Aziz M, Larsen J, Andersen PS, Pearson T, Waters AE, Foster JT, Schupp J, Gillece J, Driebe E, Liu CM, Springer B, Zdovc I, Battisti A, Franco A, Zmudzki J, Schwarz S, Butaye P, Jouy E, Pomba C, Porrero MC, Ruimy R, Smith TC, Robinson DA, Weese JS, Arriola CS, Yu F, Laurent F, Keim P, Skov R, Aarestrup FM 2012 *Staphylococcus aureus* CC398: host adaptation and emergence of methicillin resistance in livestock. mBio 3:e00305-11. doi:10.1128/mBio.00305-11. 22354957 PMC3280451

[6] Lewis HC, Mølbak K, Reese C, Aarestrup FM, Selchau M, Sørum M, Skov RL 2008 Pigs as source of methicillin-resistant *Staphylococcus aureus* CC398 infections in humans, Denmark. Emerg Infect Dis 14:1383–1389. doi:10.3201/eid1409.071576. 18760004 PMC2603104

[7] van den Broek IV, van Cleef BA, Haenen A, Broens EM, van der Wolf PJ, van den Broek MJ, Huijsdens XW, Kluytmans JAJW, van de Giessen AW, Tiemersma EW 2009 Methicillin-resistant *Staphylococcus aureus* in people living and working in pig farms. Epidemiol Infect 137:700–708. doi:10.1017/S0950268808001507. 18947444

[8] van Loo I, Huijsdens X, Tiemersma E, de Neeling A, van de Sande-Bruinsma N, Beaujean D, Voss A, Kluytmans J 2007 Emergence of methicillin-resistant *Staphylococcus aureus* of animal origin in humans. Emerg Infect Dis 13:1834–1839. doi:10.3201/eid1312.070384. 18258032 PMC2876750

[9] Larsen J, Petersen A, Sørum M, Stegger M, van Alphen L, Valentiner-Branth P, Knudsen LK, Larsen LS, Feingold B, Price LB, Andersen PS, Larsen AR, Skov RL 2015 Methicillin-resistant *Staphylococcus aureus* CC398 is an increasing cause of disease in people with no livestock contact in Denmark, 1999 to 2011. Euro Surveill 20(37):pii=21245 http://www.eurosurveillance.org/ViewArticle.aspx?ArticleId=21245. 10.2807/1560-7917.ES.2015.20.37.30021 PMC4902279 26535590

[10] van Cleef BA, Graveland H, Haenen APJ, van de Giessen AW, Heederik D, Wagenaar JA, Kluytmans AJW 2011 Persistence of livestock-associated methicillin-resistant *Staphylococcus aureus* in field workers after short-term occupational exposure to pigs and veal calves. J Clin Microbiol 49:1030–1033. doi:10.1128/JCM.00493-10. 21227986 PMC3067751

[11] Meemken D, Cuny C, Witte W, Eichler U, Staudt R, Blaha T 2008 Occurrence of MRSA in pigs and in humans involved in pig production—preliminary results of a study in the northwest of Germany. Dtsch Tierarztl Wochenschr 115(4):132–139. (In German.) 18500146

[12] Gilbert MJ, Bos MEH, Duim B, Urlings BAP, Heres Wagenaar LJA, Heederik DJJ 2012 Livestock-associated MRSA ST398 carriage in pig slaughterhouse workers related to quantitative environmental exposure. Occup Environ Med 69:472–478. doi:10.1136/oemed-2011-100069. 22544853

[13] Wertheim HF, van Kleef M, Vos MC, Ott A, Verbrugh HA, Fokkens W 2006 Nose picking and nasal carriage of *Staphylococcus aureus*. Infect Control Hosp Epidemiol 27:863–867. doi:10.1086/506401. 16874648

[14] Friese A, Schulz J, Hoehle L, Fetsch A, Tenhagen B-A, Hartung J, Roesler U 2012 Occurrence of MRSA in air and housing environment of pig barns. Vet Microbiol 158:129–135. doi:10.1016/j.vetmic.2012.01.019. 22386671

[15] Masclaux FG, Sakwinska O, Charrière N, Semaani E, Oppliger A 2013 Concentration of airborne *Staphylococcus aureus* (MRSA and MSSA), total bacteria, and endotoxins in pig farms. Ann Occup Hyg 57:550–557. doi:10.1093/annhyg/mes098. 23293050

[16] Schulz J, Friese A, Klees S, Tenhagen BA, Fetsch A, Rösler U, Hartung J 2012 Longitudinal study of the contamination of air and of soil surfaces in the vicinity of pig barns by livestock-associated methicillin-resistant *Staphylococcus aureus*. Appl Environ Microbiol 78:5666–5671. doi:10.1128/AEM.00550-12. 22685139 PMC3406131

[17] Bos MEH, Verstappen KM, van Cleef BAGL, Dohmen W, Dorado-García A, Graveland H, Duim B, Wagenaar JA, Kluytmans AJW, Heederik DJJ 2016 Transmission through air as a possible route of exposure for MRSA. J Expo Sci Environ Epidemiol 26:1–7. doi:10.1038/jes.2015.85. 25515375

[18] Wertheim HF, Melles DC, Vos MC, van Leeuwen W, van Belkum A, Verbrugh HA, Nouwen JL 2005 The role of nasal carriage in *Staphylococcus aureus* infections. Lancet Infect Dis 5:751–762. doi:10.1016/S1473-3099(05)70295-4. 16310147

[19] Frana TS, Beahm AR, Hanson BM, Kinyon JM, Layman LL, Karriker LA, Ramirez A, Smith TC 2013 Isolation and characterization of methicillin-resistant *Staphylococcus aureus* from pork farms and visiting veterinary students. PLoS One 8:e53738. doi:10.1371/journal.pone.0053738. 23301102 PMC3536740

[20] Bogaert D, van Belkum A, Sluijter M, Luijendijk A, de Groot R, Rümke HC, Verbrugh HA, Hermans PWM 2004 Colonisation by *Streptococcus pneumoniae* and *Staphylococcus aureus* in healthy children. Lancet 363:1871–1872. doi:10.1016/S0140-6736(04)16357-5. 15183627

[21] van Belkum A, Verkaik NJ, de Vogel CP, Boelens HA, Verveer J, Nouwen JL, Verbrugh HA, Wertheim HFL 2009 Reclassification of *Staphylococcus aureus* nasal carriage types. J Infect Dis 199:1820–1826. doi:10.1086/599119. 19419332

[22] Dall'Antonia M, Coen PG, Wilks M, Whiley A, Millar M 2005 Competition between methicillin-sensitive and -resistant *Staphylococcus aureus* in the anterior nares. J Hosp Infect 61:62–67. doi:10.1016/j.jhin.2005.01.008. 15893854

[23] Ghasemzadeh-Moghaddam H, Neela V, van Wamel W, Hamat RA, nor Shamsudin MN, Hussin NS, Aziz MN, Hasoani MSM, Johar A, Thevarajah S, van Belkum A 2015 Nasal carriers are more likely to acquire exogenous *Staphylococcus aureus* strains than non-carriers. Clin Microbiol Infect 21:998.e1–998.e7. doi:10.1016/j.cmi.2015.07.006. 26183299

[24] Weidenmaier C, Goerke C, Wolz C 2012 *Staphylococcus aureus* determinants for nasal colonization. Trends Microbiol 20:243–250. doi:10.1016/j.tim.2012.03.004. 22494802

[25] Nurjadi D, Lependu J, Kremsner PG, Zanger P 2012 *Staphylococcus aureus* throat carriage is associated with ABO-/secretor status. J Infect 65:310–317. doi:10.1016/j.jinf.2012.05.011. 22664149

[26] Grinshpun S, Buttner M, Mainelis G, Willeke K 2016 Sampling for airborne microorganisms, p 3.2.2-1–3.2.2-17. *In* Yates M, Nakatsu C, Miller R, Pillai S (ed), Manual of environmental microbiology, 4th edition ASM Press, Washington, DC.

[27] Goerge T, Lorenz MB, van Alen S, Hübner N-O, Becker K, Köck R 2017 MRSA colonization and infection among persons with occupational livestock exposure in Europe: prevalence, preventive options and evidence. Vet Microbiol 200:6–12. doi:10.1016/j.vetmic.2015.10.027. 26658156

[28] Islam MZ, Espinosa-Gongora C, Damborg P, Sieber RN, Munk R, Husted L, Moodley A, Skov R, Larsen J, Guardabassi L 2017 Horses in Denmark are a reservoir of diverse clones of methicillin-resistant and -susceptible *Staphylococcus aureus*. Front Microbiol 8:543. doi:10.3389/fmicb.2017.00543. 28421046 PMC5376617

[29] Frankel M, Timm M, Hansen EW, Madsen AM 2012 Comparison of sampling methods for the assessment of indoor microbial exposure. Indoor Air 22:405–414. doi:10.1111/j.1600-0668.2012.00770.x. 22299641

[30] Larsen K, Petersen JH, Budtz-Jørgensen E, Endahl L 2000 Interpreting parameters in the logistic regression model with random effects. Biometrics 56:909–914. doi:10.1111/j.0006-341X.2000.00909.x. 10985236

