# Supplementary material for: Transmission of Methicillin-Resistant Staphylococcus aureus to Human Volunteers Visiting a Swine Farm
Source: Appl Environ Microbiol. 2017 Nov 16;83(23):e01489-17. doi: 10.1128/AEM.01489-17 (PMC5691421; doi:10.1128/AEM.01489-17)
Supplement: Supplemental material [file supp_83_23_e01489-17__index.html]

Supplemental material 

# Transmission of Methicillin-Resistant Staphylococcus aureus to Human Volunteers Visiting a Swine Farm

## Supplemental material

- Supplemental file 1 -

  Numbers of MRSA-positive volunteers in the four trials (Table S1); statistics on MRSA counts in nasal swabs in relation to class variables (Table S2); air level and MRSA (Fig. S1); correlation between the nasal MRSA level at different time points after leaving the stable and the MRSA level in the air (Fig. S2); percent MRSA-positive volunteers at different time points after leaving the stable (Fig. S3).

  PDF, 311K
