# Supplementary material for: Transmission of Methicillin-Resistant Staphylococcus aureus to Human Volunteers Visiting a Swine Farm
Source: Appl Environ Microbiol. 2017 Nov 16;83(23):e01489-17. doi: 10.1128/AEM.01489-17 (PMC5691421; doi:10.1128/AEM.01489-17)
Supplement: Supplemental material [file AEM.01489-17_zam999118159s1.pdf]

SUPPLEMENTARY DATA

Supplementary Table S1

The table shows the number of MRSA-positive volunteers in the four trials. The group indicates whether the volunteers had an active (pig contact) or passive role during the stay in the farm. MRSA air indicates the geometric mean of the airborne MRSA exposure in the different groups (active, passive and days of trial). The MRSA-positive samples are indicated with green cells in the table.

ID	Trial	Day	Group	MRSA air	T=0	1 hour	2 hours	Day 1	Day 2	Day 7
1	1	1	Active		1	0	0	0	0	0
2	1	1	Active		1	1	0	1	1	0
3	1	1	Active		1	1	0	0	0	0
4	1	1	Active		1	1	0	0	0	0
5	1	1	Active		1	1	1	0	0	0
6	1	1	Active	1214	1	0	0	0	0	0
7	1	1	Passive		1	0	0	0	0	0
8	1	1	Passive		1	1	0	0	0	0
9	1	1	Passive		1	0	0	0	0	0
10	1	1	Passive		1	1	1	0	0	0
11	1	1	Passive		1	1	0	0	0	0
12	1	1	Passive	680	1	0	0	0	0	0
13	1	2	Active		1	0	0	0	0	0
14	1	2	Active		1	0	0	0	0	0
15	1	2	Active		1	0	0	0	0	0
16	1	2	Active		1	1	1	0	0	0
17	1	2	Active		1	1	1	0	0	0
18	1	2	Active	592	1	1	1	0	0	0
19	1	2	Passive		1	0	0	0	0	0
20	1	2	Passive		1	0	0	0	0	0
21	1	2	Passive		1	1	0	0	0	0
22	1	2	Passive		1	1	0	0	0	0
23	1	2	Passive		1	1	0	0	0	0
24	1	2	Passive	440	1	0	0	0	0	0
				Pos	24	13	5	1	1	0
				%	100	54	21	4	4	0
7	2	1	Active		1	1	0	0	1	1
8	2	1	Active		1	1	1	0	0	0
9	2	1	Active		1	1	1	0	0	0
10	2	1	Active		1	1	1	1	1	0
11	2	1	Active		1	1	1	0	0	0
12	2	1	Active	2557	1	1	0	1	0	0
1	2	1	Passive		1	0	0	0	0	0
2	2	1	Passive		1	1	0	0	0	0
3	2	1	Passive		1	1	1	1	1	0
4	2	1	Passive		1	0	0	0	0	0
5	2	1	Passive		1	0	0	0	0	0
27	2	1	Passive	1631	1	1	1	0	0	0
19	2	2	Active		1	0	0	0	0	0
20	2	2	Active		1	1	0	0	0	0
21	2	2	Active		1	1	1	0	0	0
23	2	2	Active		1	1	1	1	1	0
24	2	2	Active		1	1	0	0	0	0
26	2	2	Active	2499	1	1	1	0	0	0
13	2	2	Passive		1	0	0	0	0	0
14	2	2	Passive		1	0	0	0	0	0
15	2	2	Passive		1	1	0	0	0	0
16	2	2	Passive		1	1	1	0	0	0
17	2	2	Passive		1	1	1	0	0	0
18	2	2	Passive	1209	1	1	1	0	0	0
				Pos	24	18	12	4	4	1
				%	100	75	50	17	17	4
1	3	1	Passive		1	0	0	0	0	0
2	3	1	Passive		1	1	0	1	0	0
3	3	1	Passive		1	1	0	0	0	0
4	3	1	Passive		1	1	1	0	0	0
5	3	1	Passive		1	1	0	0	0	0
7	3	1	Passive		1	1	0	0	1	0
8	3	1	Passive		1	1	0	0	0	0
9	3	1	Passive		1	1	1	1	0	0
10	3	1	Passive		1	1	0	0	0	0
11	3	1	Passive		1	0	0	0	0	0
12	3	1	Passive		1	1	1	0	0	0
25	3	1	Passive	87	1	1	0	0	0	0
13	3	2	Passive		1	0	0	0	0	0
14	3	2	Passive		1	0	0	0	0	0
16	3	2	Passive		0	0	0	0	0	0
17	3	2	Passive		1	0	0	0	0	0
18	3	2	Passive		1	0	0	0	0	0
19	3	2	Passive		1	0	0	0	0	0
20	3	2	Passive		0	0	0	0	0	0
21	3	2	Passive		0	0	0	0	0	0
23	3	2	Passive		1	1	0	0	0	0
24	3	2	Passive		0	0	0	0	0	0
26	3	2	Passive		1	0	0	0	0	0
28	3	2	Passive	102	0	0	0	0	0	0
				Pos	19	11	3	2	1	0
				%	79	46	13	8	4	0
3	4	1	Passive		1	0	0	0	0	0
5	4	1	Passive		1	0	0	0	0	0
6	4	1	Passive		1	0	0	1	0	0
7	4	1	Passive		1	0	0	1	0	0
10	4	1	Passive		1	1	1	1	0	0
11	4	1	Passive		0	0	0	0	0	0
12	4	1	Passive		1	0	0	0	0	0
17	4	1	Passive		1	1	0	0	0	0
23	4	1	Passive		1	1	0	0	0	0
25	4	1	Passive		1	1	0	0	0	0
32	4	1	Passive	187	1	0	0	0	0	0
14	4	2	Passive		1	0	0	0	0	0
16	4	2	Passive		1	0	0	0	0	0
18	4	2	Passive		1	1	0	0	0	0
21	4	2	Passive		1	0	0	0	0	0
26	4	2	Passive		1	1	0	0	0	0
28	4	2	Passive		1	0	0	0	0	0
29	4	2	Passive		1	1	0	0	0	0
30	4	2	Passive		1	1	1	0	0	0
34	4	2	Passive		1	0	0	0	0	0
35	4	2	Passive		1	0	0	0	0	0
36	4	2	Passive	152	1	0	0	0	0	0
				Pos	21	8	2	3	0	0
				%	95	36	9	14	0	0
				Total	88	50	22	10	6	1
				%	94	53	23	11	6	1

Supplementary Table S2. Summary statistics on MRSA counts in nasal swabs in relation to class variables.

Variable	Level	Observations	MRSA in nasal sample (hours after leaving the stable)		
			0	1	2
All data		94	55 (18, 109)[a]	0 (0, 9)	0 (0, 0)
Gender	Male	15	55 (9, 91)	0 (0, 9)	0 (0, 0)
	Female	79	55 (18, 109)	0 (0-127)	0 (0-73)
Smoking	Yes	24	36 (5, 77)	0 (0, 9)	0 (0, 0)
	No	70	59 (27, 118)	0 (0, 9)	0 (0, 0)
Trial number	1	24	68 (45, 95)	5 (0, 9)	0 (0, 0)
	2	24	114 (36, 214)	9 (0, 18)	0 (0, 9)
	3	24	18 (0, 73)	0 (0, 9)	0 (0, 0)
	4	22	27 (9, 73)	0 (0, 9)	0 (0, 0)
Pig contact	Active	24	91 (54, 236)	9 (0, 27)	0 (0, 9)
	Passive	70	36 (9, 91)	0 (0, 9)	0 (0, 0)
MSSA carrier	Yes	55	55 (18, 118)	0 (0, 9)	0 (0, 0)
	No	39	55 (18, 91)	0 (0, 9)	0 (0, 0)

[a] CFU MRSA/ml swab fluid, median (interquartile range)

Supplementary Figure S1

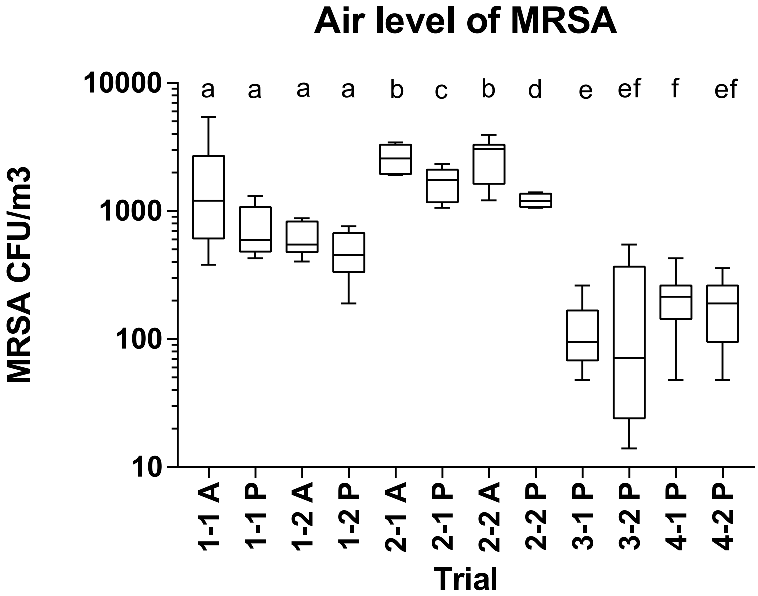

Supplementary Figure S1. Air level of MRSA (CFU/m^3) in the 4 trials, 1-1 indicates trial 1 and day 1, A=active group, P=passive group. Box and whiskers plot indicates median, 25th to 75th percentiles, and maximum and minimum values. Different letters above the graph indicate significant differences between groups (two-sided t-test, $p<0.05$).

Supplementary Figure S2

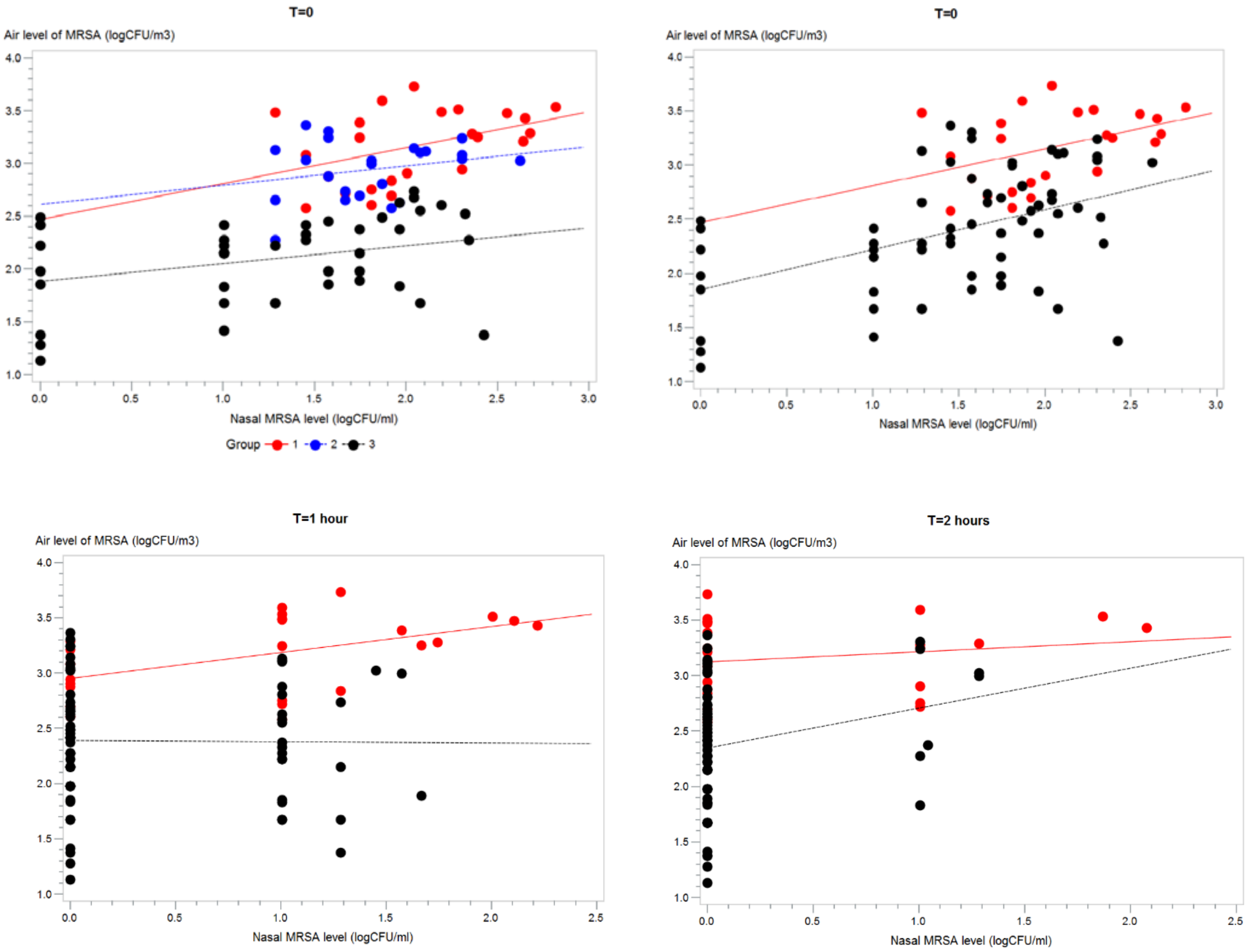

Figure S2. The correlation between the nasal MRSA level at different time points after leaving the stable and the MRSA level in the air. The data points corresponding to the active volunteers with pig contact in trials 1 and 2 are marked in red. Black points correspond to passive volunteers in all four trials. Upper left: Immediately after leaving the farm. Passive volunteers in trials 1 and 2 are marked in blue. (Regression lines, volunteers with pig contact: y=2.47 + 0.34x, R^2=0.18; passive volunteers trials 1 and 2: y=2.61 + 0.18x, R^2=0.06; passive volunteers trials 3 and 4: y=1.88 + 0.17x, R^2=0.009). Upper right: Immediately after leaving the farm with indication of active volunteers with pig contact in trials 1 and 2 and passive volunteers in trials 1-4. Lower left: 1 hour after leaving the farm. Lower right: 2 hours after leaving the farm.

Supplementary Figure S3

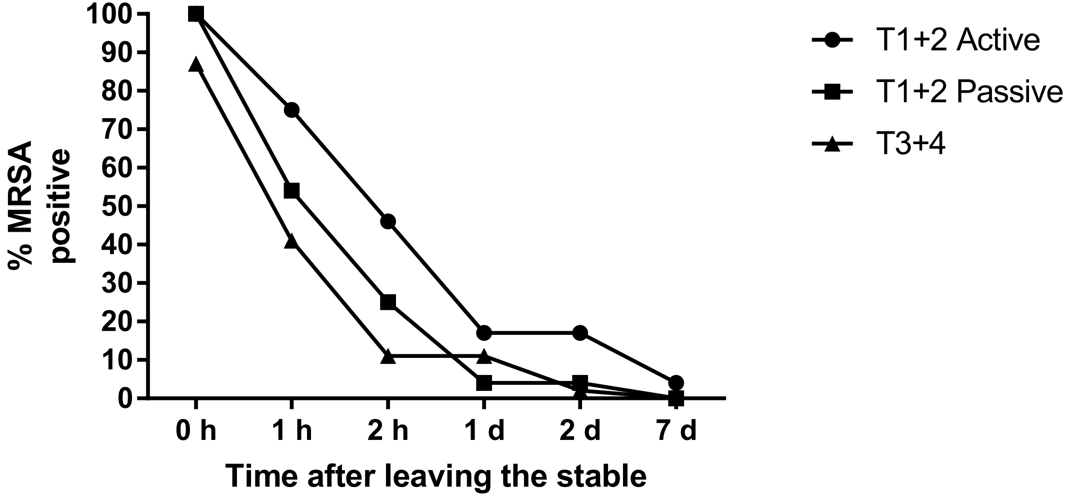

Figure S3. Percent MRSA-positive volunteers at different timepoints after leaving the stable in the following groups: Active volunteers with pig contact in trials 1 and 2 (high MRSA air level), passive volunteers in trials 1 and 2, passive volunteers in trials 3 and 4 (low MRSA air level).
